# Portuino—A Novel Portable Low-Cost Arduino-Based Photo- and Fluorimeter

**DOI:** 10.3390/s22207916

**Published:** 2022-10-18

**Authors:** Sarah Di Nonno, Roland Ulber

**Affiliations:** Chair of Bioprocess Engineering, Technical University of Kaiserslautern, 67663 Kaiserslautern, Germany

**Keywords:** portable devices, fluorimetry, photometry, 3-D printing, easy fabrication, Arduino

## Abstract

A novel portable low-cost Arduino-controlled photo- and fluorimeter for on-site measurements has been developed. The device uses LEDs as a light source and a phototransistor as a light sensor. The circuit is based on the discharge of a capacitor with the photocurrent from the phototransistor. Validation experiments for absorbance measurements were performed by measuring protein concentration using the Bradford method and measuring phosphate ions in water using a commercial test kit. The emission light of the excited fluorescent dyes rhodamine 6G and calcofluor white was measured to validate the usability of the device as a fluorescence photometer. In all validation experiments, similar correlation coefficients and limit of detection could be achieved with the portable photo- and fluorimeter and a laboratory spectrometer and fluorimeter. Real sample analysis was performed, measuring phosphate concentration in freshwater and concentration of green fluorescent protein, extracted from *Escherichia coli.*

## 1. Introduction

In the last few decades, many photometric assays were established for a wide range of parameters. The demand for inexpensive portable photometers is high, especially in areas where laboratory equipment is not available due to high cost and space requirements, or where unstable parameters must be determined quickly on-site. Therefore, a growing trend towards the use of portable analysis systems can be observed. Portable photometers have already been developed for utilization in different areas of application, for example health [1,2,3,4,5], food quality control [6,7,8,9,10,11], environmental monitoring [12,13,14,15,16,17,18,19,20] and citizen science and education [21,22].

There are two main types of portable photometric analysis systems. The first type involves the use of dispersing elements, for example the use of diffraction gratings [23] or prisms [24,25]. The light, usually coming from a broadband light source, passes through the sample, and is then dispersed into individual wavelengths by the dispersing element. This light is then often focused by lenses [4,13,25,26,27,28,29,30,31,32,33,34,35,36,37]. These optical elements are expensive and have to be arranged accurately for a precise, reproducible measurement. Furthermore, an elaborate calibration to determine the light intensity at a specific wavelength is necessary [38,39]. To make such systems accessible to a broad range of users, easy arrangement of the components and ubiquitous applicability is important. Many photometric assays are evaluated at one specific wavelength and thus render acquiring a complete absorption spectrum abundant. Because of that, light of a specific wavelength can be used, which enables the usage of an inexpensive external light sensor, for example a photodiode, phototransistor or photoresistor. Light-emitting diodes (LEDs) of a specific wavelength [10,40], or a broadband light source in combination with optical filters [12,41], can be used as single-wavelength light sources. The application of these components has the advantage that other optical elements are superfluous. Furthermore, the output value, for example the voltage at the light sensor, can be used directly without further processing. Such devices are ubiquitous usable as a stand-alone system and inexpensive. There are already some portable photometers and fluorimeters available. They can be smartphone-based [42] or controlled by a microcontroller [11,14,15,16,17,18,19,20,40,43,44,45,46,47]. Nearly all of them are to be used either as a photometer or as a fluorimeter. There are only a few devices that can be used for both applications. One of them is the smartphone spectrometer by Hossain et al. [48].

Arduino-regulated systems without implementation of a smartphone have been developed for different applications, for example for pH measurements in seawater [20]. The photometer is based on a combination of a photodiode and a transimpedance amplifier, integrated into a single component, TSL257. Another portable Arduino-based photometer, which uses the color sensor TCS230, was developed by Morais et al. [11]. Laganovska et al. developed an Arduino-controlled spectrometer which integrates a commercially available mini spectrometer [46]. Existing Arduino-based fluorimeters use UV LEDs or flashlights and emission filters in combination with photodiodes [49,50,51] or phototransistors [52]. 

By using a photodiode, phototransistor or photoresistor, the voltage at the sensor can directly be correlated with the light intensity in the measuring chamber and thus with the analyte concentration. However, there are other measurement methods. For example, there are systems which use the capacitance of LEDs for light measurements [53,54]. In these investigations, the discharge time of the p/n-junction is measured, which depends on the photocurrent. LEDs as light sensors have the advantage that they are low-cost and easily available. On the other hand, LEDs are less sensitive in comparison to a photodiode or phototransistor and their measurement wavelength is restricted, which could be considered both an advantage and a disadvantage. 

Although many portable photometers are already available, very few are designed for universal use without additional components. Furthermore, especially when used in small- or medium-sized companies and in the citizen-science sector, the reduction of costs incurred is another important point. In order to achieve these goals, a low-cost portable Arduino microcontroller-controlled photo- and fluorimeter which is based on the desklab photometer [55] was developed in this study. Due to its higher sensitivity in comparison to a photodiode, a phototransistor was used as a light sensor. However, the voltage at the phototransistor was not directly used, but a capacitor was discharged via the phototransistor instead. The discharge time of the capacitor was used as a reference for the light intensity. This measuring method enables the detection of weak light, which is especially necessary for fluorescence measurements, which were not possible without the use of this circuit. The newly developed device is called Portuino (Portable Arduino-based photo- and fluorimeter). To show the applicability of the device in different relevant fields like biotechnological industry or environmental monitoring, absorbance measurements were validated by measuring protein concentration using the Bradford method and by measuring phosphate ions in water using a commercial test kit. To validate the fluorescence measurement, the emission light of the fluorescent dyes rhodamine 6G and calcofluor white were measured in different concentrations. For all validation experiments, a comparison with the results of commercially available laboratory equipment was made. The developed system combines a stand-alone usability, a simple design, inexpensive and easy-to-use components and, in addition, a circuit that is not only simple, but also suitable for measuring weak fluorescent light. Furthermore, it can be used for variable applications in the visible and IR region due to the interchangeability of the LEDs. Therefore, the system is highly suitable for universal measurements on site. 

## 2. Materials and Methods

### 2.1. Creation of 3D Models

3D models of the measuring chamber were created with the open-source software FreeCAD (Version 0.18, Jürgen Riegel, Werner Mayer und Yorik van Havre, Germany).

### 2.2. Protein Quantification Using a Colorimetric Method after Bradford

Protein concentration measurement was done according to the Bradford method [56]. Protein standards were prepared with bovine serum albumin (BSA) (Carl Roth, Karlsruhe, Germany) in phosphate-buffered saline solution with a pH of 7.3 in a concentration range from 0 to 1000 µg mL^−1^. Coomassie protein assay reagent (Thermo Fisher Scientific, Waltham, MA, USA, ready-to-use) was used for the analysis. 30 µL of the respective protein solution was mixed with 1500 µL of the reagent solution. After 10 min, absorption at 595 nm was measured with a laboratory spectrometer (Cary 60 UV-Vis, Agilent Technologies, Santa Clara, CA, USA). A 590 nm LED was used as a light source for the absorption measurement with the Portuino. The transmitted light was measured at a 180-degree angle. With the laboratory spectrometer, the same wavelength was used. All measurements were performed in triplicate.

### 2.3. Phosphate Quantification with a Commercially Available Test Kit

Phosphate in aqueous solution was quantified with the help of a commercially available test kit, designed for aquarium water analysis (JBL PRO AQUATEST, JBL GmbH & Co., KG, Neuhofen, Germany). The kit contains reagents for the colorimetric detection of phosphate. The results can be evaluated by color comparison with printed color dots. The phosphate concentration was varied from 0.1–10 mg L^−1^ to cover the complete measurement range specified in the test kit, using a stock solution of sodium phosphate (Carl Roth, Karlsruhe, Germany) in distilled water. The standards were treated as indicated in the kit. For photometric evaluation, absorption spectra were recorded from the colored solutions in the range from 300 to 800 nm with a laboratory spectrometer (Cary 60 UV-Vis, Agilent Technologies, Santa Clara, CA, USA) and the absorption maximum was determined, which was located at 590 nm. The concentration of the analyte was evaluated with a laboratory spectrometer and the Portuino at the absorption maximum of 590 nm. With the Portuino, the transmitted light was measured at a 180-degree angle. With the laboratory spectrometer, the same wavelength was used. All measurements were performed in triplicate.

### 2.4. Quantification of the Fluorescence Dye Rhodamine 6G

The fluorescence light of rhodamine 6G (Carl Roth, Karlsruhe, Germany) was measured in ethanolic solution in a concentration range from 0 to 15 mg L^−1^. When excited at 490 nm with a laboratory fluorimeter (LS 55, PerkinElmer, Waltham, MA, USA), the dye shows an emission maximum at 552 nm. For quantification of Rhodamine 6G with the Portuino, a 490 nm UV-LED was used as light source. The emitted light was measured at a 90-degree angle to the LED. All measurements were performed in triplicate.

### 2.5. Quantification of the Fluorescence Dye Calcofluor White

The fluorescence light of calcofluor white (Sigma Aldrich, St. Louis, MO, USA) was measured in aqueous solution in a concentration range from 0 to 8 µL mL^−1^. When excited at 355 nm with a laboratory fluorimeter (LS 55, PerkinElmer, Waltham, MA, USA), the dye shows an emission maximum at 430 nm. For quantification of calcofluor white with the Portuino, a 355 nm UV-LED was used as a light source. The emitted light was measured in a 90-degree angle to the LED. All measurements were performed in triplicate.

### 2.6. Real Sample Analysis

To show applicability of the Portuino for real sample analysis, we used redox-sensitive green fluorescent protein 2 (roGFP2). The roGFP2 was provided by the cell biology group at the Technical University of Kaiserslautern. It was extracted by Escherichia coli BL21 and purified. The concentration of roGFP was measured according to the Bradford method and by fluorescence measurement. When excited at 488 nm, the protein shows an emission maximum at 507 nm [2]. Standard curves were measured, using commercially available GFP (Sigma Aldrich, St. Louis, MO, USA). For quantification of the protein with the handheld fluorimeter, a 488 nm LED was used as light source. The emitted light was measured at a 90-degree angle to the LED. Standard curves with BSA in 10 mM Tris-EDTA buffer (TE) for Bradford assay and with commercially available GFP from jellyfish Auquorea Victoria in TE were recorded.

Furthermore, phosphate measurement was performed in a freshwater sample with a commercially available test kit, as described in Section 2.3. The water sample was collected in Kistnerweiher in Neuhofen, Germany. To validate the results, phosphate was also measured by ion chromatography.

### 2.7. Data Evaluation

**Figure 1:**
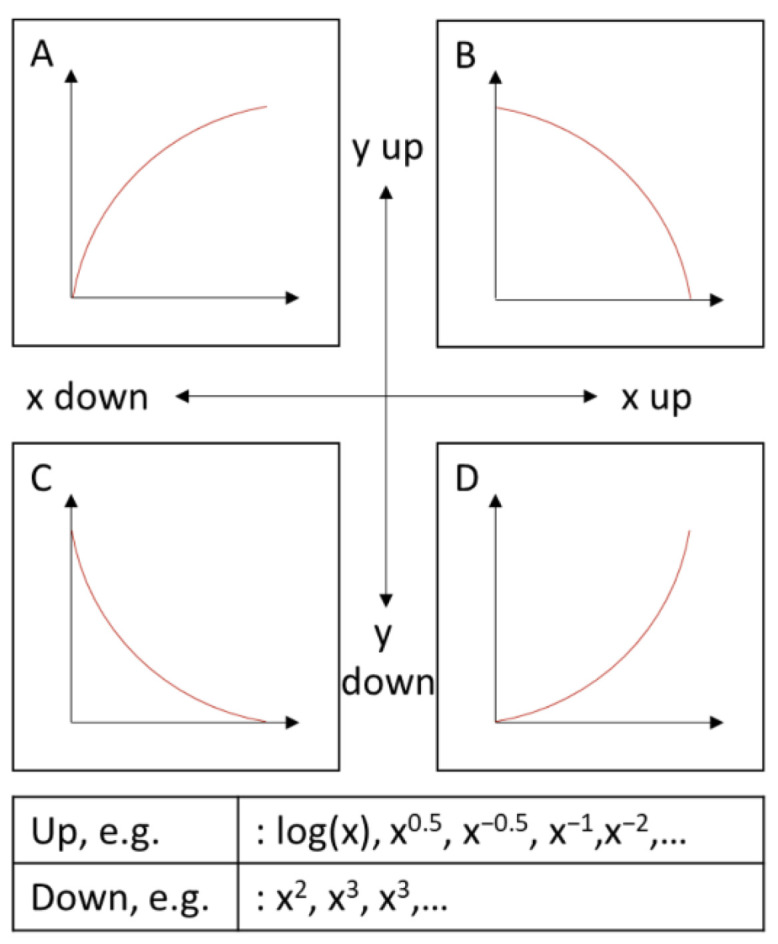
Latter of powers after Tukey [57]. Potentiation of *x*− and *y*− values for data linearization depending on the shape of the curve. A distinction is made between increasing (**A**,**D**) and decreasing (**B**,**C**) function values, and increasing (**B**,**D**) and decreasing (**A**,**C**) magnitude of slope.

The connection between the analyte concentration and the discharge time of the capacitor is not linearly correlated. This is due to the fact that there is no linear correlation between the light intensity and the photocurrent at a phototransistor. To linearize the measurement data, the ladder of power has been used [57], meaning that the data is transformed by potentiation depending on the shape of the curve (see Figure 1).

For the best linear regression, the x and y values were potentiated up or down depending on the shape of the curve. The transformation with the best linear correlation coefficient was picked to compare the data with the data of the laboratory device. 

The transformation depends on the light intensity range of the performed measurements. The larger the differences in light intensity, the more the non-linearity of the photosensor affects the measured standard series. Furthermore, the photocurrent is also affected by the wavelength of the light. The highest sensitivity of the phototransistor can be seen in the infrared region, in the range of 900 nm [58]. In the more sensitive region, the deviation from a linear standard curve will also be higher. The more the standard curve deviates from a linear one, the further the data must be transformed up or down the ladder of power. 

For automation of this transformation step, an excel script in visual basic has been developed which can be used to determine the best data transformation for the respective calibration curve (see Supplementary Data). Measurement data can directly be copied into the sheet. Care must be taken to insert six measurement data, which are needed due to the programming of the sheet. The data can also be picked with the Excel Data Streamer Add-in. After inserting a sample into the measurement chamber, a button (“Sample inserted”) can be clicked, which automatically transfers six data points to the sheet “Raw data”. After automatic or manual insertion of the data, the calculation can be started by clicking the button “Calculation”. Afterwards, the shape of the curve is determined, and transformation of the measurement data is performed. The correlation coefficient for each transformation is calculated and the transformation with the highest correlation coefficient is chosen and plotted. 

The limit of detection was calculated as described in DIN norm [59].

## 3. Results and Discussion

### 3.1. Measuring Chamber Design

In this study, a portable photo- and fluorimeter, called Portuino, has been developed. It is based on the low-cost desklab photometer, developed for educational purposes [55]. The measuring chamber of the Portuino is 3D-printed in black acrylonitrile butadiene styrene (ABS) (ABSplus^TM^, Stratasys GmbH, Frankfurt am Main, Germany).

**Figure 2:**
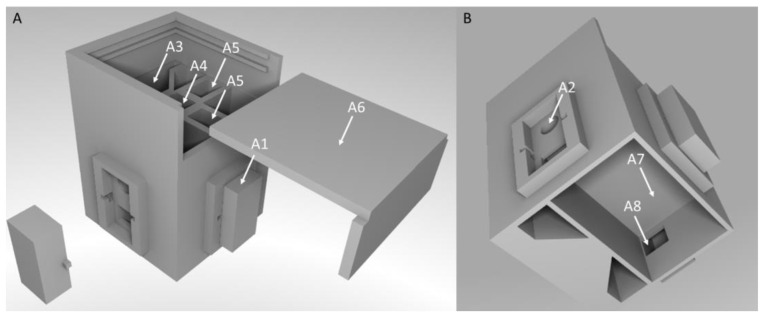
CAD-model of the developed measuring chamber of the portable photo- and fluorimeter. (**A**) = Front view of the measuring chamber of the developed photo- and fluorimeter, (**B**) = Bottom view of the measuring chamber. A1 = Cover for LED-inserts, A2 = LED insert and cutout for LED-wiring, A3 = Place for a LED board, A4 = place for a 4 mL cuvette, A5 = Pathway for light from LED to sample and from sample to phototransistor, A6 = Cover for measuring chamber, A7 = compartment for storing of the circuit board, A8 = Cutout for guiding the jumper cables from the LEDs and the phototransistor to the circuit board.

Overall dimensions of the device are (L × W × H) 90 mm × 93 mm × 95 mm. To perform absorption or fluorescence measurements, LEDs of different wavelengths can be placed in LED mounting clips (Mentor GmbH & Co., Erkrath, Germany), which can be found on three of four sides of the measuring chamber (see Figure 2 A2). The mounting clips enable an easy exchange of the components, if required. For absorption measurements, the light sensor, which is a phototransistor (SFH300, Osram GmbH, Herbrechtingen, Germany), is placed opposite from the LED. For fluorescence measurements, the LEDs can be placed at a 90-degree angle to the phototransistor to excite the sample and measure the emitted light. The cuvette is placed in the middle of the chamber (see Figure 2 A4). The LEDs and the phototransistor are connected to an Arduino Nano BLE microcontroller by jumper cables. The cables can be guided into a bottom compartment of the measuring chamber through a channel which is located below the LED-mounting clips (see Figure 2 A8). To keep out ambient light, covers were constructed which can be placed above the LEDs and the phototransistor. The wiring was soldered directly to a circuit board, which can be placed in the compartment in the bottom part of the measuring chamber (see Figure 2B A7). To ensure an even distribution of light, transparent matte labels were cut to size and placed in front of the light sensor. This measuring chamber was used for all measurements in this study.

### 3.2. Circuit Design and Arduino Program

**Figure 3:**
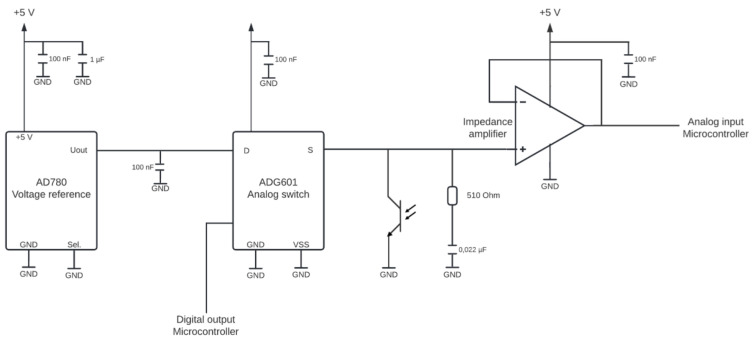
Measurement-circuit of the Portuino; reference voltage is connected to the 5 V pin of the Arduino to obtain a constant voltage supply of 3 V. An analog switch is used to turn the voltage on and off for the rest of the circuit. It is controlled by an analog pin of the Arduino. A phototransistor and a capacitor are connected to the analog switch. The voltage can be read out by an analog pin of the Arduino. To avoid current to flow through the analog pin, an impedance amplifier is connected.

To perform absorption and fluorescence measurements, a circuit based on the discharge of a capacitor was developed (see Figure 3). 

Due to the fluctuating voltage supply of the Arduino Nano, the 5V-output of the microcontroller is connected to a reference voltage (AD780AN, Analog Devices Inc., Nordwood, MA, USA, see Figure 3) to obtain a constant voltage supply of 3 V. This voltage supply can be turned on and off for the rest of the circuit by an analog switch (ADG619 BRMZ, Analog Devices Inc., Nordwood, MA, USA), which is controlled by an analog output of the Arduino Nano. A phototransistor serving as a light sensor is connected, as is a 0.022 µF capacitor which is placed in parallel to the latter. The phototransistor was chosen because of the inner amplification and the therefore higher sensitivity to lower light intensities. All other capacitors are used to stabilize the voltage in the circuit. The measuring principle is based on the discharge of the capacitor through the phototransistor. At the beginning of a measurement, the analog switch is turned on. This is done by applying a 3.3 V voltage at the logic control pin of it by the analog pin of the Arduino. This charges the capacitor. Then, the analog switch is turned off again, cutting off the voltage supply at the capacitor. Therefore, the capacitor discharges through the phototransistor. During this discharge, the applied voltage is measured using an analog input of the Arduino. The time until a voltage of 0.24 V is reached is determined. To prevent the current from flowing through the Arduino pin and forcing it to flow through the phototransistor, an impedance amplifier (OPA344PA, Texas instruments, Dallas, TX, USA) is connected between the capacitor and the Arduino pin. Because the time required to discharge the capacitor depends on the photocurrent at the phototransistor, and thus on the light intensity, this value can be used as a reference value for light absorbed or emitted by a sample. A lower light intensity at the phototransistor, resulting from a higher absorption or lower fluorescence of the sample, leads to a lower photocurrent at the phototransistor and thus to a higher measurement duration and vice versa. The Arduino is connected to a computer via USB cable and the determined measurement duration is read out via a serial monitor. The USB cable also serves as power supply for measurement chamber. It would also be possible to supply the chamber via battery, tablet (with adapter), smartphone (with adapter) or powerbank to improve field-portability. For usage without direct connection to a computer, a Bluetooth low energy (BLE) connection could be used for data readout. For this, a commercially available BLE scanner can be used, or a custom app could be programmed. 

**Figure 4:**
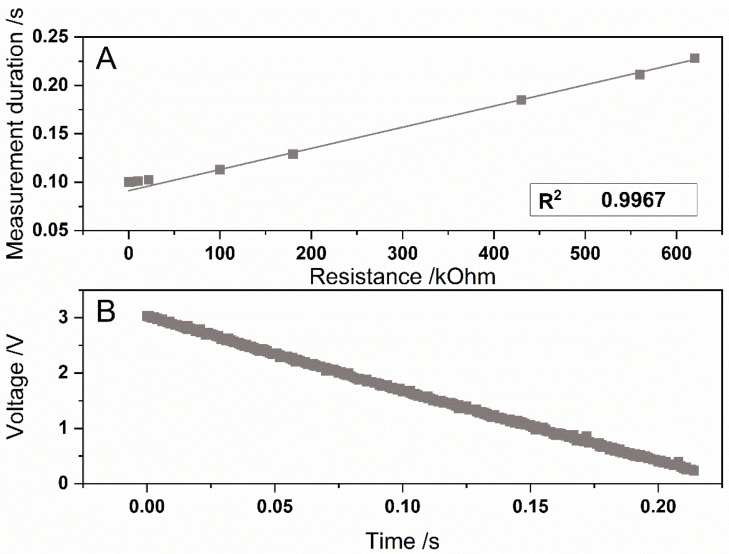
Measurement duration of the Portuino with a circuit, based on the discharge of a capacitor. The phototransistor was illuminated with a white light LED, which was connected with different series resistors with values from 0.1 to 600 kΩ. (**A**). The discharge of the capacitor was measured with an oscilloscope. The phototransistor was illuminated with the LED with a forward resistor of 560 kΩ (**B**). n = 3, and the error bars are hidden by the squares.

To test the measurement circuit, the measurement duration of the capacitor has been determined for different light intensities. For this purpose, a white-light LED was installed in the measuring chamber, and the brightness of the LED was varied by installing series resistors in the range from 0.1 to 600 kΩ (see Figure 4A). Furthermore, the discharge of the capacitor was recorded with an oscilloscope (InfiniVision DSOX2004A, Keysight Technologies, Santa Rosa, CA, USA) (see Figure 4B). When measuring the discharge of the capacitor, a linear decrease in the voltage from 3 to 0.24 V can be observed (see Figure 4B). There is a linear correlation of the forward resistor at the LED and the measurement duration from 22 to 600 kΩ., with a correlation coefficient of 0.9967. 

Considering this, care has to be taken in low absorption or high fluorescence applications because this circuit cannot measure too high light intensities. In this case, a higher forward resistor for the LED should be used.

### 3.3. Validation of the Measuring Principle of the Portuino

**Figure 5:**
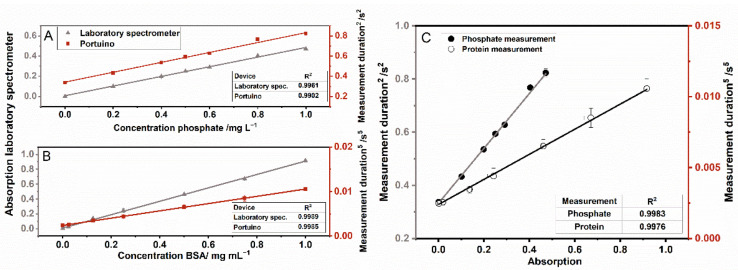
Absorption and measurement duration (squared (**A**) or to the power of 5 (**B**)) for phosphate measurement in water (**A**) and protein measurement in water with the Bradford method (**B**) with a laboratory spectrometer (grey, triangles) and the Portuino (red, squares). The absorption has been measured at 590 nm for both devices. The agreement of data was determined with the Portuino and a laboratory spectrometer (**C**). Agreement for phosphate measurement (black, circles) was performed with the help of a commercial test kit and, for the protein measurement of BSA, with the Bradford method (black, not filled circles). n = 3, the error bars are partially hidden by the symbols.

Validation of the measuring principle for absorption measurements was carried out by protein measurement with the Bradford method and phosphate measurement in water with the help of a commercial test kit. When measured with the laboratory spectrometer, a linear correlation with high correlation coefficients above 0.99 between the concentration of the analyte and the absorption could be seen for both measurements. When measured with the Portuino, a slight flattening of both measurement curves was observed in the range of high concentrations, which can be attributed to the non-linear relationship between the light intensity and the resulting photocurrent at the phototransistor [58]. Because of that, the measurement durations were squared (phosphate measurement) and potentiated with 5 (protein measurement) to get a linear standard curve (see Figure 5A,B). The fact that the transformation is different is due to the fact that a larger light intensity range from absorbance 0 to 1 is covered for protein concentration measurement compared to phosphate measurement. As described in Section 2.6, a larger deviation from a linear standard curve can be observed, which is why higher exponentiation is required, i.e., the ladder of power must be climbed further.

These transformations resulted in correlation coefficients above 0.99, which were similar to those of the measurements taken with the laboratory spectrometer. This means that the developed Portuino is suitable for absorption measurements in the investigated light ranges. The limit of detection (LOD) was calculated for both measurements. For protein concentration measurement, LOD for the Portuino and the laboratory spectrometer were 0.0086 and 0.012 mg mL^−1^. This means, that the LOD is better for Portuino in comparison with the laboratory spectrometer. For the phosphate measurement, it was the other way around. There, the LOD the Portuino and the laboratory spectrometer were 0.058 and 0.036 mg L^−1^. For both measurements, the LOD was in the same range, even if it was sometimes lower for one system and sometimes for the other. In a last step, the agreement between the Portuino measurement duration and the absorption of the laboratory device was investigated (see Figure 5C). As described above, the absorption of the analyte solution was correlated with the squared measurement duration. The agreement for all absorption measurements achieved very high correlation coefficients above 0.99. 

**Figure 6:**
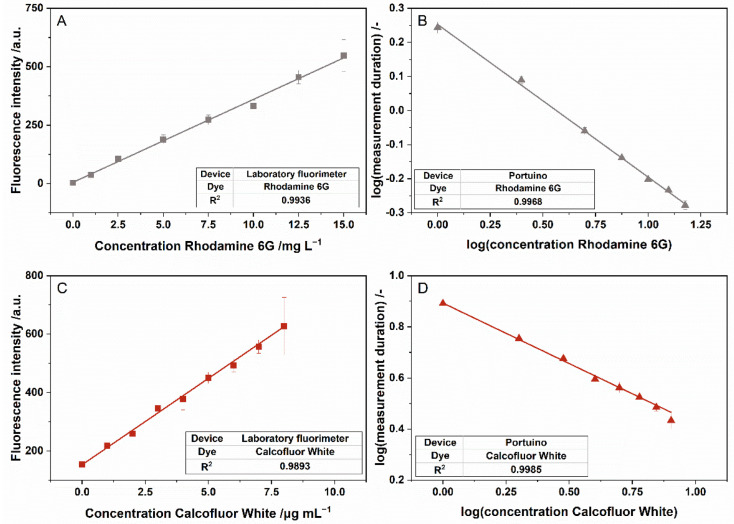
(**A**,**B**): Response of different devices for fluorescence measurement of calcofluor white. The standards were excited with light with a wavelength of 355 nm. Emitted light was measured with a laboratory fluorimeter (**A**) at a wavelength of 430 nm, and measurement duration was recorded with the Portuino (**B**). Parameter laboratory fluorimeter: Excitation slit = 10, Emission slit = 10, Gain = Low. n = 3; (**C**,**D**): Response of different devices for fluorescence measurement of calcofluor white. The standards were excited with light with a wavelength of 488 nm using the laboratory fluorimeter and with light of a wavelength of 490 nm using the Portuino. Emitted light was measured with a laboratory fluorimeter (**C**) at a wavelength of 553 nm, and measurement duration was recorded with the Portuino (**D**). Parameter laboratory fluorimeter: Excitation slit = 5, Emission slit = 5, Gain = High. n = 3.

Emission light of rhodamine 6G and calcofluor white in different concentrations was measured to validate the suitability of the Portuino for fluorescence measurements. Since no optical filters were used, care must be taken to ensure that stray light at an angle of 90 degrees did not influence the measurement. To test this effect, the absorption of two dyes were measured—a dye with an excitation wavelength in the visible range and a dye with an excitation wavelength in the ultraviolet range. The phototransistor used in this case detects light in the visible and infrared range [58], so that stray light should only affect the measurement of rhodamine 6G, if at all. When measured with the laboratory fluorimeter, there was a linear relationship between the rhodamine 6G and the calcofluor white concentration and the luminous intensity of the emitted light for all investigated concentrations (see Figure 6A,C). When using the Portuino, there was a linear relationship between the logarithmized rhodamine 6G concentration and the logarithmized measurement duration (see Figure 6B). For calcofluor white, the same transformation was used (see Figure 6D). 

Both correlations with the Portuino showed high correlation coefficients above 0.99. Furthermore, the correlation coefficient of the measurement was similar to those reached by the laboratory fluorimeter. That means that the Portuino can be used for fluorescence measurements with excitation light in the ultraviolet as well as in the visible wavelength range. Because the concentration of rhodamine 6G as well as the measurement duration had to be logarithmized for the Portuino, no agreement between the measurement data of the laboratory fluorimeter and the Portuino could be calculated. The LOD for calcofluor white measurement for the Portuino and the laboratory spectrometer were 0.52 and 0.62 µg mL^−1^. For Rhodamine 6G measurement, the LOD were 0.74 and 0.62 mg L^−1^. With this, for fluorescence measurements, there were also similar limits of detection for both devices. 

In summary, the Portuino is suitable for both absorption and fluorescence measurements. A transformation of the measurement data of the Portuino should be done due to the non-linear relationship between the light intensity at the phototransistor and the resulting photocurrent and thus, the measurement duration. Care should be taken if fluorescence measurements are performed with cloudy matrices. In this case, the light of the LED will be strayed by the sample, causing the light intensity at the phototransistor to increase. An emission filter could be required to filter stray light of the LED. For the above-shown applications, the Portuino is a simple, easy-to-build alternative for a costly and bulky laboratory device. Due to the simple design of the measurement chamber, which can be 3D printed, and the Arduino microcontroller, it can be easily built and used for point-of-care applications.

### 3.4. Real Sample Analysis

Phosphate is an important indication for the quality of freshwater. A too-high phosphate concentration leads to growth of algae and leads to eutrophication [3]. Because of that, the measurement of phosphate concentration in freshwater is important. With the Portuino, it would be possible to measure phosphate concentration directly on site without the need to bring water samples to the laboratory (see Table 1). To show applicability of the Portuino in-water analysis, phosphate is measured in freshwater. 

The results of the Portuino agree with both the laboratory spectrometric method and the ion chromatographic method. The phosphate concentration in the water sample is relatively high. For “good ecological conditions”, defined by the ordinance for the protection of surface water [4], the phosphate concentration should be under 0.22 mg L^−1^. Because the limit of quantification for phosphate measurement with the test kit is 0.15 for the Portuino and 0.10 for the laboratory spectrometer, water samples with good ecological conditions can also be measured. 

With its intense green fluorescence, GFP is an important biomarker molecule for application in medicine and diagnostics. For measurement of roGFP2 concentration after purification, fluorescence measurement was performed (see Table 1). To verify the result, protein concentration was further measured by the Bradford method.

From the results, we can see a high accordance of the Portuino in comparison to the laboratory spectrometer. Furthermore, the results of the fluorescence measurement and the protein measurement are in agreement. 

All in all, it can be seen that the Portuino is highly suitable for absorption and fluorescence measurements on-site and can replace laboratory equipment.

## 4. Future Work

In future work, there will be a smartphone application for data readout and automatic data transformation with the ladder of power. Furthermore, a higher simplicity of the device, including the use of smartphone-controlled surface-mounted LEDs on LED boards will be investigated to prevent the need for LED exchange for different measurements.

## Figures and Tables

**Table 1:** Real sample analysis with the Portuino in comparison with a laboratory spectrometer. Redox-sensitive green fluorescent protein was measured either by fluorescence measurement or protein measurement, conducted according to Bradford method. Phosphate concentration in a freshwater sample was determined using a commercially available test kit.

Method	Concentration roGFP2 with Fluorescence Measurement/mg mL^−1^	Concentration roGFP2 with Bradford Assay/mg mL^−1^	Phosphate Concentration in Freshwater/ mg L^−1^
Laboratory spectrometer	0.503 ± 0.0167	0.534 ± 0.022	0.517 ± 0.058
Portuino	0.491 ± 0.0172	0.524 ± 0.031	0.505 ± 0.031
Ion chromatography	-	-	0.471 ± 0.029

## Data Availability

Not applicable.

## Supplementary Materials

The following supporting information can be downloaded at: https://www.mdpi.com/article/10.3390/s22207916/s1, Excel Sheet S1: AutomaticDataTransformation_DiNonno_Ulber, STL-File S1: MeasuringChamber, STL-File S2: LidMeasuringChamber, STL-File S3: LidLED.
